# The diverse arsenal of type III CRISPR–Cas-associated CARF and SAVED effectors

**DOI:** 10.1042/BST20220289

**Published:** 2022-10-25

**Authors:** Jurre A. Steens, Carl Raymund P. Salazar, Raymond H.J. Staals

**Affiliations:** Laboratory of Microbiology, Wageningen University and Research, Wageningen, The Netherlands

**Keywords:** abortive infection, antiviral defense, cOA, CRISPR, nuclease, signaling

## Abstract

Type III CRISPR–Cas systems make use of a multi-subunit effector complex to target foreign (m)RNA transcripts complementary to the guide/CRISPR RNA (crRNA). Base-pairing of the target RNA with specialized regions in the crRNA not only triggers target RNA cleavage, but also activates the characteristic Cas10 subunit and sets in motion a variety of catalytic activities that starts with the production of cyclic oligoadenylate (cOA) second messenger molecules. These messenger molecules can activate an extensive arsenal of ancillary effector proteins carrying the appropriate sensory domain. Notably, the CARF and SAVED effector proteins have been responsible for renewed interest in type III CRISPR–Cas due to the extraordinary diversity of defenses against invading genetic elements. Whereas only a handful of CARF and SAVED proteins have been studied so far, many of them seem to provoke abortive infection, aimed to kill the host and provide population-wide immunity. A defining feature of these effector proteins is the variety of *in silico-*predicted catalytic domains they are fused to. In this mini-review, we discuss all currently characterized type III-associated CARF and SAVED effector proteins, highlight a few examples of predicted CARF and SAVED proteins with interesting predicted catalytic activities, and speculate how they could contribute to type III immunity.

## Introduction

CRISPR–Cas is an adaptive immune system in prokaryotes that provides sequence-specific immunity against mobile genetic elements (MGEs), such as phages, transposons and (conjugative) plasmids, although other non-immune functions have been identified as well [1,2]. Well over a decade of research has highlighted the immense diversity of these systems, as reflected by their classification that currently distinguishes two main classes, six types, and many different subtypes [3]. Nevertheless, all CRISPR–Cas systems make use of an RNA-guided protein (complex) that binds and degrades complementary MGE-derived sequences. However, type III CRISPR–Cas systems seem to be equipped with an additional layer of defense that involves the production of signaling molecules and effector proteins that respond to them.

**Figure 1.**
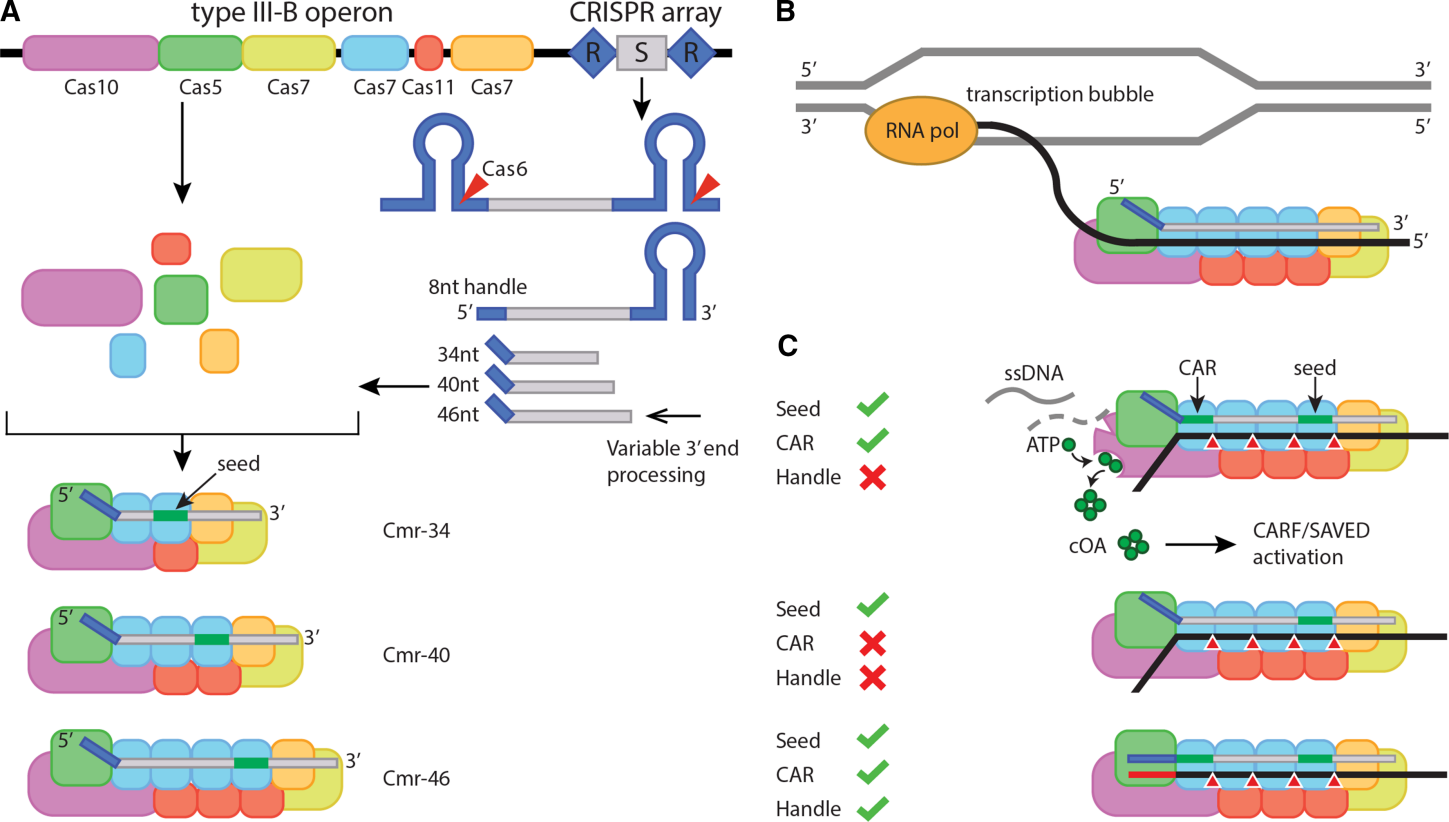
Mechanism of type III CRISPR-Cas immunity. (**A**) Overview of type III effector complex formation. Expression of the *cas* genes and processing of the CRISPR array. Repeats and spacers are indicated by blue diamonds and gray rectangles, respectively. Following endonucleolytic cleavages of Cas6, a variable 3′ end processing step of the crRNAs leads to a heterogeneous complex size population. (**B**) Biological context of type III CRISPR–Cas systems. A transcription bubble is formed when MGE-derived dsDNA is transcribed by an RNA polymerase into (m)RNA, which is subsequently targeted by type III CRISPR–Cas. (**C**) Target RNA requirements for the various activities of type III. Note that RNA cleavage only relies on complementarity in the seed region, whereas ssDNase and cyclase (cOA production) activity requires additional strict base-pairing in the CAR and no base-pairing interactions with the 5′ handle.

A typical type III system consists of several *cas* genes and a CRISPR array containing MGE-derived spacer sequences separated by repeat sequences (Figure 1A). Expression of the CRISPR array results in pre-crRNAs (pre-CRISPR RNAs) that are processed by the Cas6 protein into crRNAs [4]. These are typically further processed at their 3′ ends, resulting in mature crRNAs that start with an 8 nt repeat-derived handle at their 5′ ends and with a variable 3′ spacer-derived end [5]. Expression of the type III *cas* genes forms a complex with these crRNAs, resulting in a heterogenous population of type III ribonucleoprotein (RNP) complexes (Figure 1A).

Type III complexes are thought to operate in the context of a transcription bubble, where they bind MGE-derived transcripts complementary to the bound crRNA (Figure 1B) [6,7]. Binding is initiated at a 3′ exposed region of the crRNA called the seed sequence [8]. The variable 3′ end of the crRNA guarantees flexibility in targeting mutated RNA sequences, as the seed will be in a different location in differently sized type III complexes (Figure 1A). A seed-compliant target RNA will be bound and cleaved by the Cas7 subunits of the type III complex, cleaving it at 6-nt intervals [9–12]. Further base-pairing of the target RNA with the 5′ end, spacer-derived region on the crRNA will result in activation of the large Cas10 subunit of the complex; a region we designated as the CAR (Cas10-activating region). However, activation of Cas10 is prevented when binding self-RNAs (i.e. antisense RNA transcripts form the CRISPR array) (Figure 1C). This autoimmune protection is governed by sensing base-pairing interactions between the 5′ handle and the corresponding ribonucleotides on the target RNA. Cas10 typically contains an HD domain, capable of cleaving ssDNA substrates (potentially cleaving the exposed ssDNA regions in the transcription bubble) and a Palm domain. The Palm domain acts as a cyclase that generates cyclic oligoadenylate (cOA*_x_*, where *x* stands for the number of adenosine residues in the ring-like structure) signaling molecules from ATP [13,14]. The number of adenosine residues can vary between different type III systems, but typically are in the range of cOA_2_–cOA_6_. Subsequently, cOAs bind and allosterically activate proteins containing the appropriate sensory domains: CARF (CRISPR-associated Rossmann fold) or SAVED (second messenger oligonucleotide or dinucleotide synthetase-associated and fused to various effector domains). These sensory domains are often fused to a wide range of (predicted) catalytic domains. Over the last years, a handful of these auxiliary type III effectors have been characterized. Here, we will provide a short summary of our current understanding of these proteins (Table 1). Furthermore, we will highlight a couple of interesting examples of predicted, (non-nuclease) auxiliary type III effectors and speculate how they might contribute to type III immunity.

**Table 1.** Summary of characterized type III-associated, cOA-activatable effectors, their activating cOA species, substrate specificity, and phenotypic outcomes upon activation

Protein	Domain architecture	No. of subunits in the active form	Activating cOAs	Substrate	Phenotypic outcome	Ring nuclease activity
*Nucleases*						
Csm6	CARF-6H-HEPN	2	cOA_4_ [14,94] cOA_6_ [13,14,22,26]	ssRNA	Phage clearance [16] Dormancy [35]	Yes [21,23,94]
Csx1	CARF-HTH-HEPN	2 6 [95]	cOA_4_ [26–31]	ssRNA	Dormancy? [35]	Yes [30,31] No [27,29]
Can1	CARF-nuclease-like- CARF-PD-D/ExK	1	cOA_4_ [36]	dsDNA [36]	Phage clearance [36]	No [36]
Can2/Card1	CARF-PD-D/ExK	2	cOA_4_ [37,38]	ssDNA [37] dsDNA [38] ssRNA [37,38]	Dormancy [37] Phage clearance [37,38]	No [37,38]
NucC	PD-D/ExK	6	cOA_3_ [90,91]	dsDNA [90,91]	Cell death [90,91]	No data
*Non-nucleases*						
CRISPR-LON	Lon-SAVED	1	cOA_4_ [72]	CRISPR-T [72]	Cell death [72]	No data
TIR-SAVED	TIR-SAVED	>3	cOA_3_ [87]	NAD^+^ [87]	Cell death [87]	No data
Csa3	CARF-HTH	1	cOA_4_ [53]	dsDNA [53]	Transcriptional regulation [53]	No [53]

## CARF nucleases 

**Figure 2.**
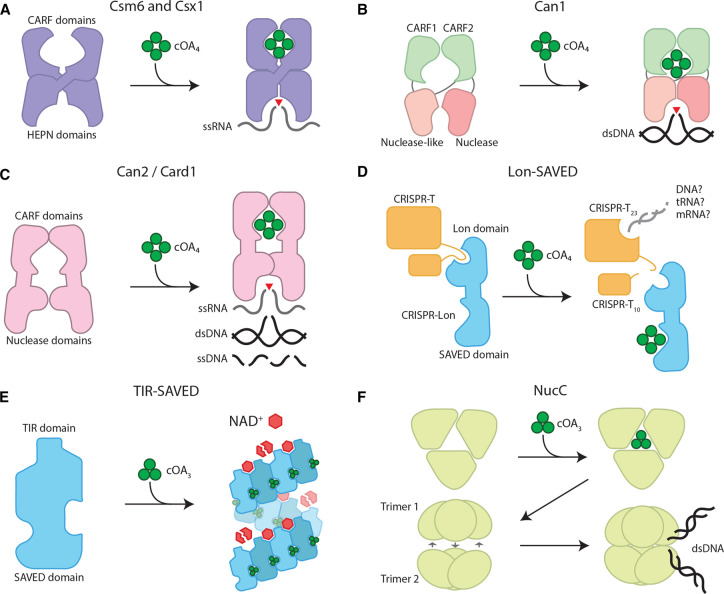
Schematic illustration of the activities of characterized type III-associated, cOA-activatable effectors. (**A**) Csm6 and Csx1 homodimers bind cOA, stabilizing them into an active form where the HEPN domains catalyze ssRNA degradation. (**B**) Can1 occurs as monomers with two CARF domains, a nuclease-like and nuclease domain. Upon cOA binding, the nuclease-like and nuclease domain form a composite active site that catalyzes dsDNA nicking. (**C**) Can2 homodimers bind cOA and shift to an active form where the nuclease domains form a composite active site that catalyzes dsDNA nicking, ssRNA and ssDNA degradation. (**D**) Lon-SAVED is initially bound to CRISPR-T, and upon cOA binding, cleaves CRISPR-T into CRISPR-T_23_ and CRISPR-T_10_. CRISPR-T_23_ then proceeds to degrade a yet unknown nucleic acid target. (**E**) TIR-SAVED forms superhelical structures upon cOA_3_ binding, forming multiple composite NADase active sites for NAD^+^ degradation. (**F**) NucC homotrimers bind cOA_3_, causing conformational changes that promote homohexamer formation, and forming dsDNA cleavage sites across the two homotrimers.

The first cOA-activatable proteins to be described were CARF nucleases, in particular Csm6 and Csx1, because they are frequently encoded in type III CRISPR–Cas operons [15] (Figure 2A). Csm6 and Csx1 were shown to function as RNases in type III interference, despite their lack of physical associations with type III complexes [10,16–20]. Investigations into how Csm6 and Csx1 are activated upon target recognition by type III complexes led to the discovery of the cOA signaling system and the function of CARF domains [13,14]. Since then, new CARF nucleases have been characterized, showing different nuclease activities aiding in defense by promiscuously degrading both self and non-self nucleic acids. Here, we will summarize the characterized CARF nucleases, their catalytic activities, and their phenotypic outcomes.

### Csm6

Among the first CARF proteins to be described was Csm6, in part due to its high prevalence in type III-A CRISPR–Cas operons [15]. These proteins have an N-terminal CARF domain and a C-terminal HEPN (higher eukaryotes and prokaryotes nucleotide-binding) domain, interspaced with an α-helical region (6H) [17]. Csm6 forms homodimers in solution to form a cOA binding pocket and a composite ribonuclease active site, in a dynamic conformational equilibrium between active and inactive forms, conferring low levels of non-specific RNase activity [16,17,21]. Upon cOA binding, Csm6 homodimers stabilize to the activated conformation formed by the histidine residues of the catalytic HEPN domain, leading to a highly-active, non-specific RNAse that cleaves single-stranded RNAs (ssRNAs) after purines (Figure 2A) [13,17,22]. Besides ssRNase activity, some Csm6 homologs have ring nuclease activity (conferred by either the CARF or HEPN domain) that cleaves bound cOAs, thereby autoregulating their activity [21,23]. *In vivo*, the contribution of Csm6 to type III immunity becomes important in situations with sub-optimal type III targeting, such as with late-expressed viral genes, mutated targets, or infrequently transcribed plasmid genes. Here, Csm6-mediated RNA degradation becomes indispensable when Cas10 HD-mediated DNA degradation is insufficient in halting MGE DNA accumulation [16,24]. During viral infections, Csm6 activity does not seem to impair cell growth, while for plasmid invasions, it causes temporary growth arrest until the plasmid is cleared [16,24]. This may be due to a high concentration of phage genomes and transcripts during infections, saturating the active Csm6, thereby reducing the impact on host transcripts.

### Csx1

Another CARF protein that was identified early on through bioinformatic analyses of type III-B CRISPR–Cas operons was Csx1 [15]. Similar to Csm6, Csx1 also has an N-terminal CARF domain and a C-terminal HEPN domain, but are separated by a helix-turn-helix (HTH) region, and forms homodimers (Figure 2A) [18,21,25,26]. Interestingly, *Sulfolobus islandicus* Csx1 (SisCsx1) has a unique structure where it forms a hexamer built from a trimer of homodimers [27]. Csx1 has non-specific ssRNase activity upon cOA binding, catalyzed by the HEPN domains. The sequence specificity of this ssRNase activity can vary between homologs: *Pyrococcus furiosus* Csx1 (PfuCsx1) cleaves after adenosines, while SisCsx1 cleaves in between two cytosine residues [18,28]. Some Csx1 homologs exhibit ring nuclease activity: PfuCsx1 can degrade cOA_4_ through the HEPN domain, while *Thermus thermophilus* Csx1 does so through the CARF domain [27,29–31]. Others, such as SisCsx1 and *S. solfataricus* Csx1, do not exhibit ring nuclease activity but seem to be dependent on dedicated ring nuclease proteins to break down the cOAs [27,32]. Interestingly, the *Marinitoga piezophila* Csx1 homolog was found fused to a dedicated ring nuclease domain, Crn2. The binding sites on both Csx1 and Crn2 domains appear to compete for cOA_4_, with the Crn2 domain having a higher affinity and faster enzyme kinetics than the Csx1 domain. This fusion may ensure that Csx1-dependent ssRNase activity is only activated when cOA_4_ levels are high enough to overcome their Crn2-dependent degradation [33]. Biologically, the importance of Csx1-related ssRNase activity in type III defense varies among different species. For example, SisCsx1 is necessary for type III-B CRISPR–Cas plasmid interference in *S. islandicus* [34]. However, in *P. furiosus*, PfuCas10 and PfuCsx1 seem to serve redundant roles in plasmid interference, where a combination of PfuCas10 HD domain mutations and either PfuCsx1 or PfuCas10 Palm domain mutations are necessary to abrogate defense [30]. The impact of Csx1-mediated RNA degradation on the fitness of the host remains to be determined, but it is suggested that it would be similar to Csm6 [35].

### Can1

Besides RNA, cOA-activatable nucleases can also degrade DNA, as seen with Can1 (CRISPR ancillary nuclease 1). Can1 appears to be limited to the genus *Thermus*, and unlike Csm6 and Csx1, Can1 operates as a monomer and contains two CARF domains separated by a nuclease-like domain and a C-terminal PD-D/ExK nuclease domain [36]. Binding of cOA to TtCan1 induces a conformational change to form a composite DNA cleavage site, formed by the nuclease-like and PD-D/ExK domains, nicking supercoiled DNA at random sites (Figure 2B). This nicking activity is believed to slow down viral replication by mediating the collapse of DNA replication forks and subsequently causing dsDNA breaks in rapidly replicating phage genomes.

### Can2/Card1

A close relative of Can1, Can2/Card1 (cOA-activated ssRNase and ssDNase 1) has a domain architecture composed of an N-terminal CARF and a C-terminal PD-D/ExK nuclease domain, and forms homodimers similar to Csm6 and Csx1 [37,38]. Upon cOA_4_ binding, two studies have shown that Can2 degrades ssRNA *in vitro* [37,38]. For DNase activity, one study showed ssDNase but not dsDNAse activity with the *Treponema succinifaciens* Can2, while another study showed progressive DNA nicking activity that eventually led to dsDNA degradation with *Sulfobacillus thermosulfidooxidans* and *Thioalkalivibrio sulfidiphilus* Can2 (Figure 2C) [37,38]. It is likely that its canonical function is DNA nicking, given two homologs are known to exhibit this activity. *In vivo*, Can2 is suggested to induce dormancy in response to phage infection and plasmid transformation [37]. Can2 is thought to introduce DNA lesions in both the host and phage genome and acts in parallel with Cas10 to eliminate target DNA [37]. In contrast with this, another study found that Can2 provided phage immunity without causing any noticeable growth defects of the host, suggesting that Can2 adequately slows down phage replication similar to Can1 [38].

## Non-nuclease CARF

Many of the currently characterized CARF proteins associated with type III immunity are nucleases, which aid in defense by promiscuously degrading both self and non-self nucleic acids. Although more research is needed, it appears that most of these systems operate as an altruistic mechanism to protect the population by inducing cell dormancy or cell death of the infected individual. Similar to other abortive infection mechanisms, there are multiple ways to induce dormancy or cell death and this is reflected by the many catalytic activities that are predicted to be associated with CARF proteins [39–48].  Here, we will discuss a few interesting examples of downstream type III effectors and speculate how they might induce dormancy or cell death.

### cOA-responsive transcriptional regulator 

**Figure 3.**
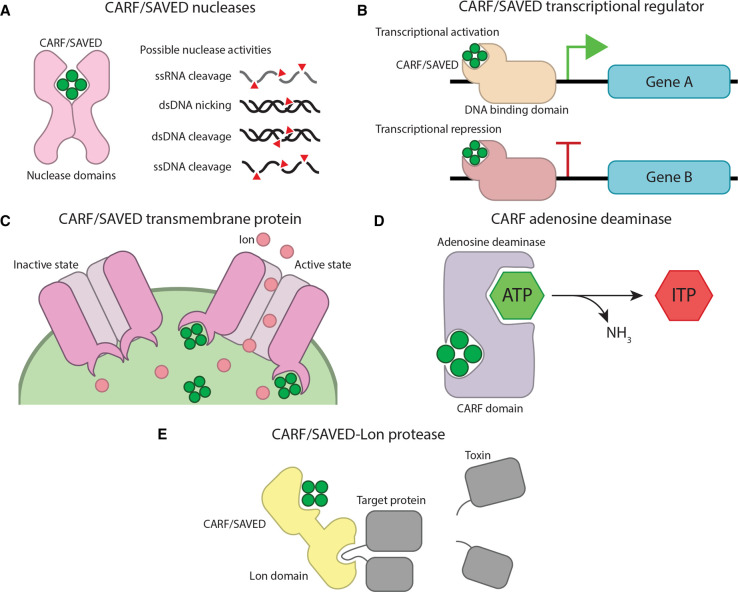
Schematic illustration of the anticipated activities of bioinformatically-predicted type III-associated, cOA-activatable effectors. (**A**) CARF or SAVED proteins with promiscuous nuclease activity, cleaving both self- and non-self nucleic acids. (**B**) CARF or SAVED proteins with DNA binding domains could enhance or repress downstream effector genes. (**C**) CARF or SAVED proteins with transmembrane domains could form pores that depolarize the membrane, depriving the cell of energy. Alternative strategies to disrupt the membrane could be employed too. (**D**) CARF proteins with predicted adenosine deaminase domains converting ATP into Inosine triphosphate (ITP), depleting cellular ATP levels. (**E**) CARF or SAVED proteins with a fused Lon protease domain liberating a toxin that kills the cell.

The only experimentally characterized non-nuclease CARF effector known to date is a transcriptional regulator, Csa3, which is often found in type I-A systems (Figure 3B) [49]. These proteins are a fusion between a CARF domain and an HTH domain, commonly involved in DNA binding and influencing expression. The complete regulatory functions of these effectors appear to be very complex, but hints at cross-talk between type III and type I systems. It has been shown that Csa3 is involved in the regulation of type I CRISPR adaptation, as well as providing a feedback loop to type III interference [50–54]. Furthermore, Csa3-mediated activation of DNA repair genes has been demonstrated, indicating that the network of gene regulation by type III associated effectors might not be constricted to CRISPR-related genes [55]. 

### Transmembrane CARF effectors

*In silico* analyses indicated that many CARF proteins are fused to a transmembrane (TM) domain [39]. One mechanism could be that these CARF-TM proteins form ion channels in the membrane upon activation by cOAs (Figure 3C). Subsequent depolarization of the membrane would be a means to disrupt many processes in the cell, eventually resulting in altruistic cell death, akin to some superinfection exclusion systems encoded on prophages [40]. Alternatively, CARF-TM activation could also result in complete mechanical disruption of the membrane, as seen in other abortive infection systems [47,56]. 

### CARF adenosine deaminase

Although not as widespread as the CARF-TM fusions, some CARF proteins have an adenosine deaminase (ADA) domain. These enzymes typically convert adenosines into inosine residues, and some non-CARF-associated ADAs have important housekeeping roles in prokaryotes, such as the editing of tRNAs [57]. Activated CARF-ADA could, therefore, act by dysregulating these processes to induce cell death. However, the deamination of nucleotides could also be leveraged to deplete the cell of ATP, similar to a strategy that is employed by other phage defense systems (Figure 3D) [48].

### CARF-Lon effectors

The active domain of a family of housekeeping proteases, the Lon domain, also seems to be adapted for type III-mediated phage defense, as demonstrated by the bioinformatically predicted CARF-Lon fusions [39]. Canonical Lon proteases are involved in the degradation of misfolded and abnormal proteins as well as certain regulatory proteins [58–61]. In the context of phage defense, we speculate that the CARF-Lon protein, when activated by cOA, could either cleave essential host protein targets or acts as an aggressive promiscuous protease, both geared towards killing the host (Figure 3D). 

## SAVED effector proteins 

In archaea and bacteria, cyclic oligonucleotide-based antiphage signaling system (CBASS) immunity systems are widespread, providing a diverse arsenal of anti-phage defense tools [62–66]. Typically, these systems encode a cGAS/DncV-like nucleotidyltransferase (CD-NTase) protein, responsible for sensing the presence of phage and the subsequent synthesis of a second messenger molecule [67,68]. The messenger molecules resemble signaling molecules of type III defense but can contain a variety of nucleotide moieties and different linkages between them. Upon recognition by CD-NTase-associated protein (Cap) effectors, an elaborate immune response is initiated that can lead to cell death. SAVED is a common sensing domain for these Cap proteins and has long been predicted to be involved in type III immunity, but was until recently not experimentally demonstrated [39,69].  Although this sensing domain has limited sequence similarity to CARF domains, it is thought to be a highly divergent version of CARF, fused to a variety of effector domains [39,68,70].

### SAVED nucleases

Similar to the abovementioned CARF-nuclease fusions, a large array of different domains predicted to confer non-specific (ribo)nucleases are commonly found in SAVED proteins (Figure 3A) [39]. Notably, the SAVED-HNH fusion proteins appear to be a common example. Of note, this domain, named after the catalytic residues, is also responsible for sequence-specific target cleavage in some type II CRISPR–Cas systems [3,71].

### Lon-SAVED

The first example of a connection between SAVED and type III CRISPR–Cas, a Lon-SAVED protease, was recently demonstrated and revealed a new mechanism by this system to aid in defense [72]. The Lon-SAVED effector (CRISPR-Lon) contains a C-terminal SAVED sensing domain, consisting of two CARF-like domains, fused to a N-terminal Lon protease domain. Binding of a cOA_4_ messenger molecule induces an allosteric change in the protein that activates this effector (Figure 2D). Interestingly and in contrast with canonical Lon proteases, CRISPR-Lon appears to have a specific target protein, CRISPR-T. The 32 kDa CRISPR-T protein is cleaved by activated CRISPR-Lon into two fragments (∼23 and ∼10 kDa). The ∼23 kDa fragment bears structural similarity to MazF, which is a toxin known to cleave specific rRNA, mRNA and tRNA molecules, leading to abortive infection [73,74]. The triggering of a toxin/cell-death signal by a protease is something observed in both prokaryotes and eukaryotes and seems to be an evolutionarily conserved strategy for inducing cell death [75,76]. It is, therefore, anticipated that similar type III CRISPR–Cas protease-mediated defense strategies will be uncovered, acting through different protease-like toxins that perturb essential cellular targets. A similar strategy was found to be deployed by a type III-E associated caspase effector, which is activated via protein:protein interactions between it and the type III complex rather than cOA signaling [77–79].

### TIR-SAVED

The Toll/interleukin-1 receptor (TIR) domain is widely found in all domains of life [80–84]. In humans, this domain is often present in Toll-like receptors to mediate signaling for innate immunity. In response to binding their ligand(s), TIR domains of certain immune receptors in plants synthesize a signaling molecule to induce cell death [82]. In Thoeris, a bacterial anti-phage defense system, TIR domains are responsible for the production of nicotinamide adenine dinucleotide (NAD) derived signaling molecules which in turn allosterically activate a TIR domain-containing enzyme that aggressively depletes NAD^+^ to arrest cell growth [85]. The depletion of NAD^+^ is a strategy that is also employed by a prokaryotic short Argonaute immune system upon the detection of invading DNA [86]. 

The modularity of known CBASS systems and their interplay with CRISPR–Cas defense is highlighted by a recent study on a TIR-SAVED effector protein [87]. The CBASS system it originated from generates cOA_3_ messenger molecules. These are bound by TIR-SAVED and mediate its multimerization, forming composite active sites to degrade NAD^+^ (Figure 2E). TIR-SAVED can induce cell death *in vivo* when placed in the context of a type III system by replacing the canonical Csm6 CARF ribonuclease by the TIR-SAVED effector. This demonstrates interchangeability between CBASS and CRISPR–Cas defense systems, but natural examples of type III CRISPR–Cas systems in combination with the NucC nuclease and TIR-SAVED effectors exist as well [39]. The widespread usage of TIR domains in defense systems across the domains of life can be seen as proof that several eukaryotic immune systems originated from an ancestral prokaryotic anti-phage system [83]. 

### Transmembrane SAVED effectors

Similar to CARF effectors, many SAVED proteins containing a TM domain have been predicted bioinformatically [3]. A similar mechanism as described for the CARF-TM fusions could be employed by this type of SAVED effector (Figure 3B). Although rather speculative, an exciting possibility arises that these TM type III effectors position the sensory SAVED domain on the outside of the cell, hinting at intercellular signaling. Signaling the presence of infection to others in the population can be seen in other CRISPR–Cas systems as a means to strengthen the immune response [88,89].

## NucC, a non-CARF and non-SAVED effector

cOA-activatable effectors are not limited to CARF and SAVED proteins, as exemplified by NucC. NucC (nuclease, CD-NTase associated) is a CBASS-associated protein that has also been found in 31 type III CRISPR–Cas loci [90]. In both CBASS and type III systems, NucC forms homotrimers with three active sites on the outer edge. Upon binding of cOA_3_, pairs of NucC homotrimers bind to form homohexamers, juxtaposing pairs of partial active sites between the two homotrimers and forming dsDNase active sites (Figure 2F). This results in double-stranded breaks on dsDNA with two-base 3′ overhangs [90]. *In vivo*, in both CBASS and type III CRISPR–Cas systems, NucC appears to act through an abortive infection mechanism whereby its activation causes the complete destruction of the host chromosome, culminating in cell death [90,91]. This can be a beneficial characteristic of this nuclease to overcome phage escape strategies, such as the ability of type III-associated NucC to overcome jumbo phage infections in *Serratia* [91]. These phages protect their DNA after injection using a proteinaceous nucleus inside the host [92]. Instead of targeting phage DNA, NucC degrades the host genome and thereby kills the host to prevent phage progeny (abortive infection).

## Perspectives

- Type III CRISPR–Cas systems are sophisticated multilayered immune systems, aiming to clear invading MGEs by cleaving target RNAs complementary to the guide RNA, but will also produce cOA signaling molecules to activate its second layer of defense, mediated by CARF and SAVED proteins.
- CARF and SAVED proteins have a plethora of catalytic activities associated with them, most of which seem to be geared towards killing the infected host (and thereby preventing viral progeny) to provide population-wide immunity; a mechanism known as abortive infection. Collateral damage observed in other CRISPR–Cas systems indicates that this strategy is not limited to type III [2].
- Most CARF proteins characterized to date are sequence-unspecific (ribo)nucleases that induce cell death or dormancy by cleaving both self and non-self nucleic acids. However, bioinformatic analyses have shown that many other CARF and SAVED proteins are fused to other catalytic domains (proteases, deaminases, NADases, etc.). If and how these different activities contribute to type III mediated defense and what effect they will have on the fitness of the host will be an interesting challenge for the future.
- An unresolved issue is how adaptive type III CRISPR–Cas systems that elicit abortive infection select for interference-proficient spacers in nature. To resolve this issue, more work on investigating the fate of clonal subpopulations in spatially structured ecological niches is required [93].

## Abbreviations

ADA: adenosine deaminase
Can: CRISPR ancillary nuclease
Cap: CD-NTase-associated protein
CAR: Cas10-activating region
Card: cOA-activated ssRNase and ssDNase
CARF: CRISPR-associated Rossmann fold
CBASS: cyclic oligonucleotide-based antiphage signaling system
CD-NTase: cGAS/DncV-like nucleotidyltransferase
cOA: cyclic oligoadenylate
CRISPR–Cas: clustered regularly interspaced short palindromic repeats-CRISPR-associated genes
HEPN: higher eukaryotes and prokaryotes nucleotide-binding
HTH: helix-turn-helix
MGE: mobile genetic element
NAD: nicotinamide adenine dinucleotide
NucC: nuclease, CD-NTase associated
SAVED: second messenger oligonucleotide or dinucleotide synthetase-associated and fused to various effector domains
TIR: Toll/interleukin-1 receptor
TM: transmembrane
